# In Vitro and In Vivo Antidiabetic Potential of Monoterpenoids: An Update

**DOI:** 10.3390/molecules27010182

**Published:** 2021-12-29

**Authors:** Lina T. Al Kury, Aya Abdoh, Kamel Ikbariah, Bassem Sadek, Mohamed Mahgoub

**Affiliations:** 1 Department of Health Sciences, College of Natural and Health Sciences, Zayed University, Abu Dhabi P.O. Box 144534, United Arab Emirates; 2 School of Medicine, Royal College of Surgeons in Ireland—Bahrain, Muharraq P.O. Box 15503, Bahrain; 18253687@rcsi.com (A.A.); 18206441@rcsi.com (K.I.); 3 Zayed Center for Health Sciences, College of Medicine and Health Sciences, United Arab Emirates University, Abu Dhabi P.O. Box 144534, United Arab Emirates; bassem.sadek@uaeu.ac.ae; 4 Pharmacy Department, SEHA, Abu Dhabi Health Services, Abu Dhabi P.O. Box 144534, United Arab Emirates; Momahgoub@seha.ae

**Keywords:** diabetes mellitus, anti-diabetic drugs, monoterpenes

## Abstract

Diabetes mellitus (DM) is a chronic metabolic condition characterized by persistent hyperglycemia due to insufficient insulin levels or insulin resistance. Despite the availability of several oral and injectable hypoglycemic agents, their use is associated with a wide range of side effects. Monoterpenes are compounds extracted from different plants including herbs, vegetables, and fruits and they contribute to their aroma and flavor. Based on their chemical structure, monoterpenes are classified into acyclic, monocyclic, and bicyclic monoterpenes. They have been found to exhibit numerous biological and medicinal effects such as antipruritic, antioxidant, anti-inflammatory, and analgesic activities. Therefore, monoterpenes emerged as promising molecules that can be used therapeutically to treat a vast range of diseases. Additionally, monoterpenes were found to modulate enzymes and proteins that contribute to insulin resistance and other pathological events caused by DM. In this review, we highlight the different mechanisms by which monoterpenes can be used in the pharmacological intervention of DM via the alteration of certain enzymes, proteins, and pathways involved in the pathophysiology of DM. Based on the fact that monoterpenes have multiple mechanisms of action on different targets in in vitro and in vivo studies, they can be considered as lead compounds for developing effective hypoglycemic agents. Incorporating these compounds in clinical trials is needed to investigate their actions in diabetic patients in order to confirm their ability in controlling hyperglycemia.

## 1. Introduction

Diabetes mellitus (DM) is a chronic metabolic condition characterized by endocrine abnormalities and persistent hyperglycemia [1,2,3]. DM can be classified into several types based on the etiology, clinical manifestations, and management; however, persistent high levels of glucose and hyperlipidemia are the major common aspects between all the major types of DM [4,5,6,7]. Due to its complexity, DM and its complications remain a substantial medical problem. Most of the available conventional drugs, despite their therapeutic benefits, can produce some undesirable side effects and are expensive. Therefore, the search for antidiabetic drugs, specifically plant-based medicine, gains importance due to their potential therapeutic effects. Recently, several phytochemicals have been shown to possess antidiabetic properties, and many efforts have been carried out to elucidate their possible antidiabetic mechanisms. Monoterpenes are a group of secondary plant metabolites that are widespread in nature and have significant hypoglycemic effect, which has been well-documented in several experimental studies [8,9,10,11]. The aim of this review is to overview the activities and the underlying mechanisms by which monoterpenes exhibit their antidiabetic effects against DM. The novelty of this study stems from the fact that it highlights the most recent findings on the mechanisms of monoterpenes in in vitro and in vivo studies using animal models, which in turn provides a window of opportunity for future research in this field. 

## 2. Diabetes Mellitus and Its Pathogenesis

DM is classified into four main subtypes including type 1 diabetes mellitus (T1DM), type 2 diabetes mellitus (T2DM), gestational diabetes mellitus [12], and maturity-onset diabetes of the young (MODY) [13]. T1DM, also known as insulin-dependent DM, occurs due to the destruction of insulin-producing β-cells in the pancreas via autoimmune mechanisms. Consequently, this leads to the scantiness of insulin levels and hence patients require exogenous insulin supply [14,15,16,17]. T2DM, however, is characterized by what is known as insulin resistance (IR) [18,19]. On the contrary, gestational diabetes is an acute form of DM affecting pregnant women as a result of perturbations in the levels of different hormones such as estrogen, progesterone, and cortisol [4,20]. MODY, the rarest type of DM, results from mutations in the genes involved in glucose metabolism [5,21]. 

Under normal conditions, the molecular events involved in insulin signaling are initiated by glucose oxidation and its facilitated diffusion into β-cell by glucose transporter 2 (GLUT2), the main transporter of glucose in the intestine, pancreas, liver, and kidney. Following the entry of glucose, it is phosphorylated by glucokinase enzyme into glucose-6-phospahte (G6P) which is considered the sensor for glucose in the pancreatic β-cell and plays a central function in insulin secretion. Further metabolism of G6P produces ATP, which inhibits ATP-sensitive K^+^ channels and results in membrane depolarization and calcium influx through L-type voltage-dependent calcium channels. The rise in intracellular calcium stimulates insulin release into the bloodstream [22].

**Figure 1:**
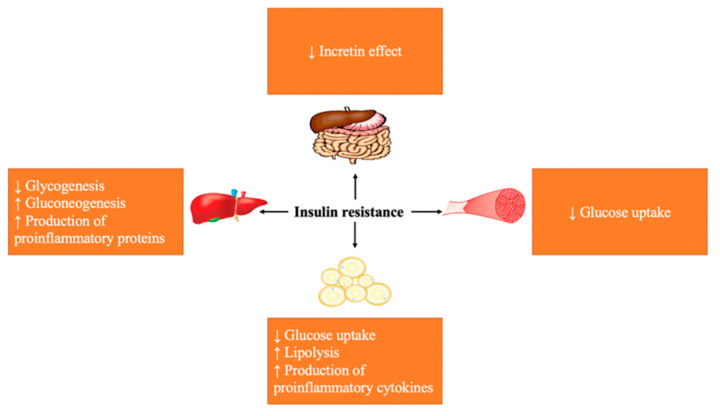
Effects of insulin resistance on body organs and tissues.

Unlike T1DM, pancreatic production of insulin in T2DM may remain intact. However, the action of insulin on various body organs is the cardinal pathological condition which occurs due to IR, causing impaired glucose uptake by muscle tissue, inhibition of hepatic glucose synthesis, and increased lipolysis (Figure 1) [23,24]. Typically, pancreatic β-cells counteract for the diminished effect of insulin through increasing the release of insulin to reverse hyperglycemia; however, as IR worsens, this compensatory mechanism becomes less effective. Consequently, the insulin-producing capacity of the pancreas progressively diminishes, leading to the eventual loss of pancreatic β-cells mass, apoptosis, and complete loss of insulin production [25,26,27,28]. It is important to mention that insulin sensitivity and/or activity is physiologically regulated by various factors such as circulating hormone levels, plasma lipids, adipokines, and their respective signaling pathways [29,30,31]. The interaction between those pathways and the insulin pathway tunes the sensitivity and activity of insulin.

After a meal, approximately two-thirds of the ingested glucose is utilized by skeletal muscles through an insulin-dependent mechanism. Following its binding to its receptor, insulin enhances the migration of the glucose transporter 4 (GLUT4) from the intracellular compartment to the plasma membrane, where it facilitates the uptake of glucose [32,33]. Insulin binds to the α-subunit of the insulin receptor (INSR) and causes phosphorylation of tyrosine residues in the β-subunit, which is followed by the recruitment of different substrates such as insulin receptor substrate-1 (IRS-1), insulin receptor substrate-2 (IRS-2), and phosphoinositide 3-kinase (PI3K) [34]. In addition to the utilization by skeletal muscle, a large portion of glucose is absorbed from the intestines and taken up by hepatocytes to be converted into glycogen via the action of insulin [35]. Upon binding to its receptor, insulin causes a cascade of phosphorylation for several downstream proteins that regulate various metabolic pathways such as gluconeogenesis, glycogen synthesis, glycogenolysis, and lipid synthesis [36]. These metabolic processes are finely tuned by the actions of insulin and glucagon, where insulin promotes glucose storage and glycogen synthesis, while glucagon promotes hepatic glucose production and glycogen breakdown [35,37,38]. It is important to mention that development of hepatic IR impairs insulin response in the hepatocytes, which results in the inhibition of glycogen synthesis and the increase in hepatic gluconeogenesis, lipogenesis, and synthesis of proinflammatory proteins such as C-reactive protein (CRP). This can lead to an ongoing inflammatory state in the liver that consequently exacerbates IR [39,40]. 

Postprandially, insulin binding to its receptor in adipose tissue facilitates the uptake of glucose by GLUT4. This subsequently activates glycolysis, from which glycerol-3-phosphate (G3P) is produced and esterified with other fatty acid- forming triacylglycerols that act as a source of energy in the fasting state [41]. Adipose IR impairs the actions of insulin and can therefore lead to impaired uptake of free fatty acids from the blood, enhanced lipolysis, and impaired glucose uptake [42]. At the molecular level, it was found that adipose IR causes activation of a defective form of AKT that impairs the translocation of GLUT4 to the membrane and activates lipolytic enzymes, which consequently worsens hyperglycemia. On the contrary, high levels of free fatty acids in the bloodstream can lead to their accumulation in other organs such as the liver, which eventually affects insulin sensitivity and hepatic gluconeogenesis and worsens T2DM [39,41].

Adipose tissue has a dynamic endocrine role and releases different proteins known as adipokines [43,44]. It has been reported that an increase in adipose tissue size and/or mass is associated with fibrosis, hypoxia, macrophage-mediated inflammation, and pathologic vascularization [45]. High-fat diet can stimulate mitochondrial proteins and transcription factors that cause adipose tissue inflammation and dysfunction [46]. The changes in the size of adipocytes and the infiltration of immune cells induce the production of proinflammatory cytokines such as tumor necrosis factor-α (TNF-α) and interleukins (IL-6 and IL-1β). This causes a chronic state of inflammation known as metabolic inflammation which plays a significant part in IR and T2DM, consequently [47].

In addition to the above-mentioned events, two types of incretins, namely glucagon-like peptide 1 (GLP-1) and glucose-dependent insulinotropic peptide (GIP) are released from the intestine after meals to stimulate pancreatic insulin secretion [14,48,49]. These peptides have a short duration of action due to their deactivation via the dipeptidyl peptidase-4 (DPP-4) enzyme [50]. While both GLP-1 and GIP share the same effect on insulin secretion [51,52,53], only GLP-1 can suppress the secretion of glucagon [54,55] and exhibit growth-factor-like effects on pancreatic β-cells, stimulating insulin gene expression and insulin biosynthesis [56,57]. For this reason, GLP-1 arose as an important pharmacological target in the formulation of antidiabetic therapies via mimicking its effect [58,59]. In T2DM, the action and the level of incretins are adversely affected [60], and the glucose-dependent secretion of insulin is reduced in the fed state [61,62]. The pancreas becomes less responsive to GIP, while it remains responsive to GLP-1 [63]. This could be justified by either an uprise in the expression of DPP-4 or a reduction in the expression of GIP and GLP-1 receptors [64,65].

## 3. Conventional Hypoglycemic Agents

**Figure 2:**
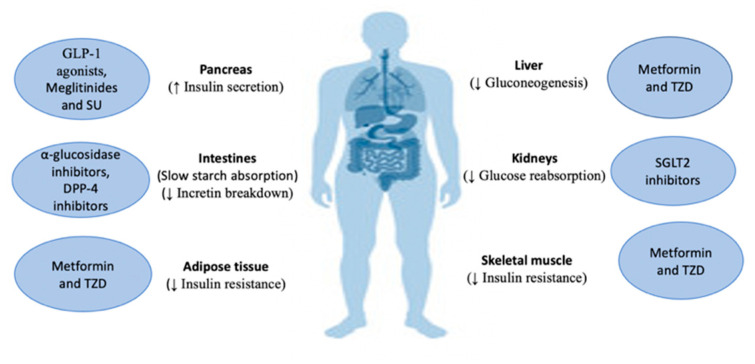
Mechanisms of action of hypoglycemic agents: dipeptidyl peptidase-4 (DPP-4); glucagon-like peptide 1 (GLP-1); sodium-glucose co-transporter-2 (SGLT2); sulfonylureas (SU); thiazolidinediones (TZD).

Up to this day, different pharmacologic agents have been used to limit the effects of hyperglycemia in diabetes. The mechanisms by which hypoglycemia is achieved include stimulation of insulin secretion by sulfonylureas and meglitinides, stimulation of peripheral glucose absorption by thiazolidinediones and biguanides, delay of carbohydrate absorption from the intestine by alpha-glucosidase, and reduction of hepatic gluconeogenesis by biguanides. Combining lifestyle modifications (such as diet and exercise) and using hypoglycemic agents is important to achieve long-term metabolic control and to protect against health complications caused by DM. Several studies investigated this treatment modality and showed the superiority of combining both lifestyle changes and pharmacological agents in the management of T2DM over using antidiabetic agents alone [66,67,68,69,70,71,72]. Various injectable and oral therapeutic agents have been developed and used clinically in the management of T2DM, each of which has a unique mechanism of action that targets different pathological events occurring in T2DM [18,73,74] (Figure 2). For example, metformin exhibits its effects by inhibiting hepatic gluconeogenesis [75,76,77], reducing insulin resistance in skeletal muscle and adipose tissue and promoting the release of GLP-1 [78]. Furthermore, metformin lowers plasma lipid levels by acting on the peroxisome proliferator-activated receptor (PPAR-α) pathway.

Sulfonylureas (SU) are insulin secretagogues that exert their action directly on the pancreas by inhibiting ATP-dependent potassium channels on the pancreatic β-cells, which causes cell depolarization and increases intracellular Ca^2+^ levels, resulting in insulin secretion [74]. Additionally, they inhibit the breakdown of lipids in the liver and decrease insulin clearance [79]. Although SU are associated with weight gain and hypoglycemic attacks, they remain one of the most widely used agents in the management of T2DM due to their high efficacy in reducing blood glucose levels [80]. Another group of insulin secretagogues are meglitinides, which work through a mechanism similar to that of SU [81]. However, they cause less weight gain and hypoglycemic attacks in comparison to SU, which makes them an ideal alternative for patients complaining of these side effects [74]. Thiazolidinediones (TZD) are a group of drugs that exert their effects by acting on the liver, skeletal muscle, and adipose tissue where they reduce insulin resistance and improve tissue sensitivity to insulin through the activation of PPAR-γ [82]. Moreover, TZD can also act on another isoform of PPAR-α which accounts for its lipid-lowering properties. TZD administration results in multiple actions such as maintaining pancreatic β-cell integrity, decreasing the levels of inflammatory cytokines, and increasing the levels of a protein known as adiponectin that is released from adipose tissue, causing an overall improvement in insulin sensitivity [27,83]. Alpha-glucosidase inhibitors such as acarbose, work by inhibiting the enzyme α-glucosidase, which functions via the conversion of oligosaccharides into monosaccharides in the small intestines [84]. Acarbose has a similar structure to that of oligosaccharides, which allows it to compete for the binding site in the enzyme. As a result, a delay in the postprandial absorption of glucose is achieved along with a reduction in hyperglycemia. The enzyme DPP-4 is responsible for the breakdown of incretin. Due to its physiological function, it arose as a target for the management of T2DM [85]. In 2007, sitagliptin was approved by the Food and Drug Administration (FDA), making it the first DPP-4 inhibitor. By inhibiting DPP-4, the action of incretins is prolonged, which in turn improves insulin secretion, reduces glucagon secretion, and decreases the rate of nutrient absorption into the bloodstream [86,87]. As mentioned previously, GLP-1 agonists became available for use in the management of T2DM in 2005 when the first GLP-1 agonist was approved by the FDA [88,89]. GLP-1 and GLP-1 agonists bind to the GLP-1 receptor on pancreatic β-cells and inhibit ATP-activated K^+^ channels through activation of protein kinase A (PKA)-dependent pathway [90,91]. Sodium glucose co-transporter-2 inhibitors are the newest class of oral hypoglycemics that exert their action on renal tubules by suppressing the sodium glucose co-transporter-2, which reduces the reabsorption of glucose and enhances its excretion [28,92,93,94,95,96].

## 4. Monoterpenes in Diabetes

**Figure 3:**
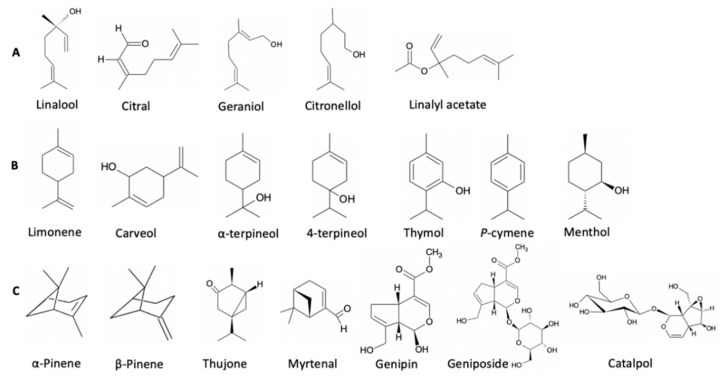
(**A**) Acyclic monoterpenes, (**B**) monocyclic monoterpenes, (**C**) bicyclic monoterpenes.

Despite the management of diabetes via the use of conventional pharmacological agents, DM and its complications remain a substantial medical problem. The majority of synthetic oral glucose-lowering drugs exhibit significant side effects and are expensive. Therefore, there has recently been a shift of interest toward exploring natural plant products for their pharmacological effects, including the treatment of diabetes. Monoterpenes are an important group of secondary metabolites that belong to the terpenoids family of natural products and have been recognized for their wide range of cellular and molecular activities that could potentially underlie their positive therapeutic index. Furthermore, their low cost, availability, low undesirable side effects, and better safety profile mark them as promising source for synthesizing new and effective agents to treat DM. For example, monoterpenes such as thymol and carvacrol are common ingredients of food and therefore, not expected to have undesirable effects. Monoterpenes are composed of two isoprene units with a general molecular formula of C_10_H_16_ and frequently contain one double bond in their structures [11]. Monoterpenes exist in over 30 known skeletons and can be classified into three subgroups: acyclic, monocyclic, and bicyclic monoterpenes [97] (Figure 3). Common examples of the acyclic form include linalool, citral, and geraniol, while important representatives of monocyclic monoterpenes include limonene, carveol, and menthol. 

According to the size of their second ring, bicyclic monoterpenes can be classified into three classes. The first ring in each class is a six-membered ring while the second can be either a three (e.g., thujone), four (e.g., α- and β-pinene), or five (e.g., borneol and camphor)-membered ring. Their hydrophobic property along with their small molecular weight makes them the major components found in nearly all essential oils. Studies have reported that both natural monoterpenes and their synthetic derivatives have a vast array of pharmacological actions including anti-diabetic, hypocholesterolemic, antioxidant, antibacterial, anti-inflammatory, anti-cancer, antihistaminic, and analgesic actions [98,99,100]. This review highlights the potential therapeutic effects of monoterpenes in DM.

### 4.1. Acyclic Monoterpenes

#### 4.1.1. Linalool

Linalool (3,7-dimethyl-1,6-octadiene-3-ol) is one of the main monoterpenoids found in herbal essential oils of many plants such as lavender (*Lavandula* spp.), which is known for its antiarrhythmic effect. Furthermore, linalool is a main component of rose (*Rosa* spp.), basil (*Ocimum basilicum*), neroli oil (*Citrus aurantium*) [101] and found in both green and black tea. Linalool has been implicated in aroma and flavoring [102]. Previous studies have reported potent antioxidant and antidiabetic activity of linalool [103,104]. Linalool was found to have favorable effects on glucose metabolism in animal models of diabetes [105]. Garba et al., 2020 investigated the antidiabetic action of lemongrass tea in T2DM model of rats. The findings of this study have shown that consumption of lemongrass reduced blood glucose levels by 60.3% [106]. Linalool, one of the main active ingredients of lemongrass, was shown to attenuate hyperglycemia and its associated complications [105]. The results were supported by higher glucose tolerance in lemongrass-treated diabetic rats in comparison to control diabetic rats which could be associated with the high content of linalool [106]. 

The enzymes α-amylase and α-glucosidase are accountable for the breakdown of carbohydrates and for the hydrolysis of starch into glucose pre-absorption. A reduction in hyperglycemia postprandially is due to the inhibition of α-amylase, which retards carbohydrate digestion and decreases glucose levels in the blood [107]. Therefore, inhibition of carbohydrate digestion in the gastrointestinal tract by α-amylase is one of the approaches to treat diabetes. Previous studies have demonstrated that lemongrass could effectively inhibit α-amylase and α-glucosidase activity [108]. For example, α-amylase inhibitory activity of the essential oil of lemon grass, for which linalool is the main active constituent, was found to be fifteen times higher compared to the currently used glucose lowering drug acarbose [109], while the inhibitory activity of methanol extract of lemon grass on α-glucosidase was more than 50% [108]. 

The uptake of glucose using rat diaphragm is a commonly used method to measure peripheral utilization of glucose in in vitro studies [110]. Linalool demonstrated dose-dependent uptake of glucose. At a concentration of 3 mM, linalool causes an increased uptake of glucose that is almost equivalent to two units of insulin. Furthermore, linalool was found to reduce oxidative stress and stimulate the activity of the antioxidant enzymes, catalase, and superoxide dismutase [105].

#### 4.1.2. Citral

Citral (3,7-dimethylocta-2,6-dienal) is a combination of the *cis* and *trans* isomers geranial and neral, and can be found in all citrus fruits and lemon grass (*Cymbopogon citratus*) [111]. *Cymbopogon citratus* has been used over the years in Indian traditional medicine as a sedative and to treat headaches and fever [111]. Citral was shown to reduce hyperglycemia and attenuate diabetes-associated complications in earlier studies [112]. A study has reported that citral exhibits a 45.7% inhibitory effect on α-amylase at a concentration of 10 mM [98]. In streptozotocin-treated rats, citral inhibited mammalian α-amylase, with an IC_50_ of 120 μM, and reduced α-amylase levels in vivo. In addition, citral treatment caused a moderate decrease in postprandial glucose and normalized blood lipid profile [112]. Due to their direct influence on the control of energy balance via glucose uptake, lipogenesis, and lipolysis, 3T3-L1 adipocytes are among the most commonly used cell culture models to study obesity and T2DM. In 3T3-L1 adipocytes, 1 μM of citral was found to suppress the proliferation by 29.2% [98]. The results of these studies suggest that citral could be a potential antihyperlipidemic agent in diabetes. It is worth noting that several antihyperlipidemic agents such as bile acid sequestrants exhibited a promising glucose lowering activity. Such agents target bile acid receptors, which play a crucial role in metabolic diseases [113,114]. In fact, colesevelam, a bile acid sequestrant, caused a significant reduction in HbA1c and fasting plasma glucose levels. Additionally, it resulted in an increase in the levels of circulating incretins when used by patients with T2DM [115,116]. Furthermore, other types of lipid lowering agents such as fibrates [117] and cholesterol absorption inhibitors such as ezetimibe [118] have also been reported to improve glycemic control and insulin activity through unknown mechanisms.

Citral inhibits the retinaldehyde dehydrogenase enzyme and therefore raises adipose tissue retinaldehyde levels, leading to the inhibition of adipogenesis, increase in metabolic rate, reduction of weight gain, and enhanced tolerance to glucose. Treating 6-week-old male Sprague–Dawley rats with citral (10, 15, and 20 mg/kg bodyweight for 28 days) caused a noticeable reduction in the increase of body weight. Additionally, citral-treated rats had lower fasting glucose levels, enhanced glucose tolerance and metabolic rate, and lower abdominal fat accumulation [119].

Supporting the above findings, a study recently conducted by Mishra et al., 2019 revealed that citral has antidiabetic as well as dyslipidemic activities. In streptozotocin-induced diabetic rats on a high-fat diet, citral application significantly diminished glucose levels in the blood and increased insulin levels in the plasma. Moreover, citral ameliorated oxidative markers along with anti-oxidative enzymes of the pancreas, liver, and adipose tissue, and regulated the activity of the glucose-metabolic enzymes in the liver [120]. 

#### 4.1.3. Geraniol

Geraniol (3,7-dimethylocta-trans-2,6-dien-1-ol) is an acyclic monoterpene alcohol found in many aromatic plants including *Cinnamomum tenuipilum* and *Valeriana officinalis*. In traditional medicine, geraniol has been used to treat many ailments including diabetes [121]. In streptozotocin-induced diabetic rats, application of geraniol for 45 days led to a significant dose-dependent increase in insulin levels and reduction in glycated hemoglobin, HbA1c. Furthermore, geraniol was found to ameliorate the function of the enzymes responsible for the metabolism and utilization of glucose. Geraniol additionally improved glycogen content in hepatocytes and preserved the histology of hepatic and pancreatic β-cells in streptozotocin-induced diabetic rats [122].

A recent work conducted by Kamble et al., 2020 demonstrated for the first time the efficacy of geraniol in inhibiting GLUT2 [123]. Inhibition of GLUT2 in the intestine, liver, and kidney plays a critical role in lowering glucose levels in the blood. Moreover, the inhibition of GLUT2 on pancreatic β-cells is anticipated to guard β-cells from glucotoxicity.

Prolonged treatment with geraniol (29.37 mm/kg body weight twice a day for 60 days) enhanced the lipid profile and HbA1c levels [123]. In another study, 1 μM of geraniol resulted in the suppression of 3T3-L1 pre-adipocyte proliferation by 19.9% [98]. It is clear from these findings that geraniol could be a novel drug in treatment of DM due to the fact that it is effective in lowering blood glucose and improving lipid profile.

#### 4.1.4. Citronellol

Citronellol (3,7-dimethyl-6-octen-1-ol) is a linear monoterpene alcohol naturally found in about 70 essential oils, with abundance in *Cymbopogon nardus* (L.) and citrus oil [124,125]. *Cymbopogon nardus* was previously used in Chinese medicine to treat rheumatism, fever, and digestive problems [126]. Although citronellol has been reported to possess strong antioxidant, anti-inflammatory, anti-cancer, and cardioprotective properties [127,128], its role in diabetes is not well-investigated.

Oral administration of citronellol (25, 50, and 100 mg/kg bodyweight for 30 days) attenuated the hyperglycemia in streptozotocin-induced diabetic rats. Citronellol improved insulin, hemoglobin, and hepatic glycogen levels and decreased HbA1c concentration. Furthermore, there was a near to normal restoration of the altered activity of carbohydrate metabolic enzymes as well as hepatic and kidney markers. Citronellol supplement preserved the histology of hepatic cells and pancreatic β-cells in streptozotocin-treated rats [124]. 

Glucose uptake plays an important role in the control of plasma glucose level, thus directly influencing glucose tolerance. Treating 3T3-L1 adipocytes with 1 μM of citronellol exerted about 16% enhancement in glucose uptake [98]. 

#### 4.1.5. Linalyl Acetate

Linalyl acetate (3,7-dimethylocta-1,6-dien-3-yl acetate) is the primary constituent of lavender (*Lavandula angustifolia*) which is known in folk medicine for its sedative effect [129]. It is also a main component of *Salvia sclarea* oil [130]. It has been shown that linalyl acetate possesses an anti-inflammatory effect and can restore endothelial function in rats after oxidative stress [104,131]. To date, the reported therapeutic effects of linalyl acetate in hyperglycemia are scarce. Treatment with 100 mg/kg linalyl acetate was more efficient in correcting serum glucose than the antidiabetic drug metformin in streptozotocin-induced diabetic rats. In addition, the observed cardiovascular protective and metabolic stabilization effects of linalyl acetate could be attributed to its antioxidative and anti-inflammatory properties, its increase in AMP-activated protein kinase expression, and its suppression of excess serum NO [132]. The antidiabetic effects of acyclic monoterpenes are summarized in Table 1.

### 4.2. Monocyclic Monoterpene

#### 4.2.1. Limonene

Limonene [1-methyl-4-(1-methylethenyl)-cyclohexene] is the main constituent of oils extracted from orange, lemon, grapefruit, and other citrus plants. It is also frequently used as a food additive, and a constituent of soaps and perfumes. As per the Code of Federal Regulations, D-limonene is classified as a safe flavoring compound [133].

Limonene was shown to reduce hyperglycemia and attenuate diabetes-associated complications in earlier studies [105,134]. Inhibition of protein glycation is known to improve secondary complications in diabetes. In streptozotocin-induced diabetic rats, limonene (100 µM) revealed 85.61% reduction in protein glycation [105]. In a study conducted by Joglekar et al., 2013, limonene was shown to inhibit protein glycation by 56.3% at a concentration of 50 μM. Furthermore, BSA was used as a model protein in PatchDock studies, which have shown that limonene has the ability to bind to the key glycation sites IB, IIA, and IIB sub domains. It was concluded that limonene is a powerful inhibitor of protein glycation that exhibits its effects by a novel mechanism of stabilization of protein structure through hydrophobic interactions [135]. In 3T3-L1 adipocytes, 1 µM of (R)-(+)-limonene stimulated both the uptake of glucose and breakdown of fats. It also upregulated glucose transporter 1 (GLUT1) expression and suppressed adipose triglyceride lipase (ATGL). (R)-(+)-limonene (at mM range) also suppressed both α-amylase and α-glucosidase; however, such outcome was weak [98]. 

In oral streptozotocin-induced diabetic rats, administration of D-limonene (50, 100 and 200 mg/kg body weight) for 45 days resulted in a significant drop in plasma glucose and HbA1c levels. Furthermore, it resulted in a decrease in the activity of the enzymes involved in gluconeogenesis, including glucose 6-phosphatase (G6Pase) as well as fructose 1,6-bisphosphatase. On the contrary, D-limonene inhibited liver glycogen as well as the activity of the glycolytic enzyme glucokinase in diabetic rats. Such antidiabetic effects were proportional with glibenclamide [136]. These findings support the potential antihyperglycemic activity of D-limonene reported in the literature.

Limonene, alone and in combination with linalool, was found to reduce oxidative stress and intensify the activity of the antioxidant enzymes catalase and superoxide dismutase [105]. The shielding role of D-limonene against diabetes and its complications was demonstrated by Bacanlı et al., 2017 [134]. In streptozotocin-induced diabetic rats, D-limonene treatment (50 mg/kg body weight for 28 days) caused a remarkable reduction in DNA damage, glutathione reductase enzyme activity, and malondialdehyde (MDA) levels in the plasma. In addition, it caused a significant increase in the levels of glutathione and the activities of catalase, superoxide dismutase, and glutathione peroxidase. Overall, lipid levels and liver enzymes were adjusted in diabetic rats [134]. 

#### 4.2.2. Carveol

The monoterpene carveol [2-methyl-5-(prop-1-en-2-yl)cyclohex-2-en-1-ol] is a component of the essential oils of *Cymbopogon giganteus* [137], *Illicium pachyphyllum* [138], and *Carum carvi* [139]. It is also present in orange peel, caraway seeds, and dill. Carveol is broadly used in perfumes, soap, and shampoos [140] and has several pharmacological activities including antioxidant, anticancer [141], antimicrobial [99], and anti-inflammatory [142] effects. In addition, carveol has a low toxicity profile [143]. 

Recently, the antidiabetic capacity of carveol was evaluated in in vivo, in vitro, and in silico studies. In alloxan-induced diabetic rats, carveol caused concentration- and time-dependent decrease in the level of glucose in the blood. Carveol (394.1 µM/kg) amended oral glucose tolerance surplus in rats and attenuated the HbA1c level and mediated hepatoprotective and anti-hyperlipidemic effects [8]. In in vitro assay, carveol inhibited α-amylase activity in a dose-dependent manner. In addition, carveol revealed binding affinity toward different targets associated with diabetes. In silico evaluation showed that carveol had maximum binding affinity (lowest energy value) toward the sodium-glucose co-transporter, intermediate binding affinity against fructose-1,6-bisphosphatase, and lowest affinity toward phosphoenolpyruvate carboxykinase (PEPCK) and glycogen synthase kinase-3β (PEPCK) [142]. The results of this study support the antidiabetic potential of carveol.

#### 4.2.3. Terpineol

Terpineol [2-(4-methyl-3-cyclohexen-1-yl)-2-propanol] is a main constituent of Marjoram (*Origanum majorana*) and Maritime pine (*Pinus pinaster*) [144]. Terpineol is widely used in food and household products. Although the antioxidant and anti-inflammatory effects of terpineol have been documented previously, studies highlighting its direct antidiabetic effects are very limited. In a recent study, in vitro α-amylase enzymatic assay has shown that both α-terpineol and its structural isomer 4-terpineol caused an inhibition in its enzymatic activity by 33% (IC_50_ 1.01 ± 0.0221 mg/mL) and 40% (IC_50_ 0.838 ± 0.0335 mg/mL) respectively, when tested individually at a concentration of 0.670 mg/mL [145]. Furthermore, terpineol was recently reported to upregulate insulin sensitivity and lessen serum levels of pro-inflammatory cytokines in rats fed with high fat diet [146].

#### 4.2.4. Thymol

Thymol (2-isopropyl-5-methylphenol), a natural phenolic monoterpenoid obtained mainly from the Thymus species (*Trachyspermum ammi* L. Sprague) [145], has been used in folk medicine to treat various ailments such as diabetes and respiratory disorders [147]. Thymol is a potent antioxidant and scavenger for hydroxyl radicals and superoxide anions [148]. Earlier studies on thymol have reported antimicrobial [149], anti-inflammatory [150], as well as anticancer potential [151].

In obese murine model fed with high fat diet, thymol treatment decreased body weight gain as well as visceral fat-pad weight. Additionally, an overall reduction in the levels of lipids was observed. The enzymes alanine aminotransferase, aspartate aminotransaminase, and lactate dehydrogenase were also reduced. Furthermore, thymol decreased the levels of glucose and leptin, decreased serum lipid peroxidation, and improved the levels of antioxidants [152]. Similarly, in mice fed with high-fat diet, thymol treatment (20, 40 mg/kg daily) significantly reversed body weight gain and peripheral insulin resistance [153]. Saravanan and Pari, (2015) tested the antihyperglycemic and antihyperlipidemic effects of thymol in diabetic C57BL/6J mice fed with high-fat diet. Daily intragastric application of thymol (40 mg/kg body weight) for 5 weeks caused a significant decline in plasma glucose, HbA1c, insulin resistance, and leptin. Moreover, it lowered the levels of plasma triglycerides, total cholesterol, free fatty acids, and low-density lipoprotein. On the other hand, thymol increased high density lipoprotein cholesterol. In addition, thymol significantly decreased hepatic lipid content including triglycerides, free fatty acids, total cholesterol, and phospholipids [154]. More recently, Saravanan and Pari [155] have shown that thymol possesses a protective role against diabetic nephropathy in C57BL/6J mice. Thymol hindered the activation of transforming growth factor-β1 (TGF-β1) and vascular endothelial growth factor (VEGF). In addition, it caused a substantial increase in the antioxidants, inhibited lipid peroxidation markers in erythrocytes and kidney tissue and reduced the lipid accumulation in kidney [156]. 

Supporting these results, a more recent study has shown that in streptozotocin-treated diabetic rats, 20 and 40 mg/kg thymol significantly reduced the levels of creatinine, low-density lipoprotein cholesterol, and hepatic enzymes including aspartate aminotransferase and alanine aminotransferase. Furthermore, the antioxidant enzyme status was also modulated after treatment with thymol [157]. Such findings indicate that thymol may possess promising protective and anti-diabetic activity.

The antidiabetic and antioxidant properties of *Thymus quinquecostatus* Celak, of which thymol is the main active constituent, were investigated. High level of thymol in *T. quinquecostatus* shows the potential of this plant as a crude drug and dietary health supplement. The ethyl acetate fraction of the methanol crude extract of *T. quinquecostatus* possessed a strong antioxidant activity. In hexane fraction, α-glucosidase inhibitory activity was positively correlated with the amount of thymol, indicating that thymol is the primary source for antioxidant and antidiabetic activity of *T. quinquecostatus* [158].

The inhibitory activity of thymol (5.0 mg/mL) and its synergistic effect with *p*-cymene (2.5 mg/mL) were linked to their antioxidant property by reducing the formation of advanced glycation end products. Based on spectroscopic and electrochemical methods, in combination with molecular docking study, it was found that the binding affinity of thymol with bovine serum albumin is greater than glucose. Furthermore, thymol had a protective effect toward arginine or lysine modification, indicating that it has an anti-glycation property [9].

#### 4.2.5. *p*-Cymene

*p*-Cymene [1-methyl-4-(1-methylethyl) benzene] is an essential oil component found in over 100 plants, including *Cuminum cyminum* and thyme. Due to its use as an intermediate in the industrial manufacturing of food flavoring, fragrances, herbicides, and medications, *p*-Cymene possesses a significant commercial role [159,160]. *p*-Cymene is the biological precursor of carvacrol and has a structure that is similar to thymol [161]. Earlier studies have reported antioxidant [162] and anti-inflammatory [160] activity of *p*-cymene. In high fat diet-treated adult NMRI mice, *p*-cymene (20 mg/kg) led to an apparent drop in blood glucose levels as well as alanine aminotransferase and alkaline phosphatase. Additionally, a slight alteration was detected in lipid profile. Interestingly, the effects of *p*-cymene were comparable with metformin [163]. Similar findings were also observed with thymol [152]. 

In streptozotocin-induced diabetic rats, administration of *p*-cymene (20 mg/kg body weight for 60 days) was found to lower HbA1c. Biophysical studies showed that *p-*cymene can inhibit glycation-mediated conversion of α-helix to β-pleated sheet structure of bovine serum albumin. Interestingly, it produced antiglycation effects when used in concentrations that were 10–20 times less than the known protein glycation inhibitors, without exhibiting any toxic effects [164].

#### 4.2.6. Menthol

Menthol [5-methyl-2-(propan-2-yl) cyclohexan-1-ol], is a component of essential oils such as eucalyptus and lemongrass and is responsible for the characteristic smell and flavor of *Mentha longifloia* that has been used traditionally in Asia for the treatment of respiratory illnesses. Menthol occurs in four isomers namely, (+)- and (−)-menthol, (+)- and (−)-neomenthol, (+)- and (−)-neoisomenthol, and (+)- and (−)-isomenthol; however, (−)-menthol (L-menthol) is the major form that exists in nature [165]. Menthol is used to treat several conditions including the common cold and other respiratory conditions, gastrointestinal disorders, as well as musculoskeletal pain [166]. In streptozotocin-nicotinamide induced diabetic rats, application of menthol (25, 50, and 100 mg/kg/body weight) and glibenclamide (600 μg/kg/body weight) for 45 days caused a significant reduction in the overall levels of blood glucose and HbA1c. It also resulted in an increase in the level of plasma insulin, liver glycogen, and total hemoglobin. Furthermore, menthol ameliorated glucose-metabolizing enzymes, protected hepatic and pancreatic islets, and suppressed pancreatic β-cells apoptosis in diabetic rats. The later effect was coupled with a rise in anti-apoptotic Bcl-2 expression and a fall in pro-apoptotic Bax expression [167]. In a more recent study, acute oral (200 mg/kg) and topical administration (10% *w*/*v*) of menthol to high fat-fed diabetic mice were found to increase serum glucagon concentration 2 h after administration. Furthermore, chronic oral administration of menthol (50 and 100 mg/kg/day) for 12 weeks and topical application (10% *w*/*v*) prevented high fat diet-induced weight gain, adipose tissue hypertrophy, liver triacylglycerol depletion, and insulin resistance. The consequent metabolic changes of menthol in the liver and adipose tissue imitated the role of glucagon. In the liver, an increase in glycogenolysis and gluconeogenesis was observed. Additionally, the thermogenic activity of adipose tissue was boosted. Interestingly, in mature 3T3L1 adipocytes, treatment with the serum of menthol-treated mice improved the markers of energy expenditure, which was blocked following the administration of the non-competitive glucagon receptor antagonist, L-168,049. This effect shows that the increase in serum glucagon induced by menthol administration is responsible for the rise in energy expenditure [168]. The antidiabetic effects of monocyclic monoterpenes are summarized in Table 2.

### 4.3. Bicyclic Monoterpenes

#### 4.3.1. α- and β-Pinene

α-pinene [(1S,5S)-2,6,6-trimethylbicyclo[3.1.1]hept-2-ene ((−)-α-Pinene)], is a major component of the volatile oil extract of the herb *Foeniculum vulgare* (fennel). Earlier studies have reported anti-inflammatory, hypoglycemic, and hepatoprotective effects of fennel [169]. In alloxan-induced diabetic mice, α-pinene evoked hypoglycemia at the 2nd and 24th hours of treatment. In addition, it was reported that α-pinene possesses a strong anti-inflammatory effect at a concentration of 0.50 mL/kg [169]. 

β-Pinene [6,6-dimethyl-2-methylidenebicyclo[3.1.1]heptane Pin-2(10)-ene] is found in numerous essential oils which possess antioxidant potential. It is one of the key constituents of the hexanic extract of *Eryngium carlinae,* commonly referred to as the “frog herb”, which has been shown to reduce hyperglycemia and hyperlipidemia and exert antioxidant activity in diabetic rats [170,171].

*Pistacia atlantica* has been proposed to have a protective effect against conditions associated with oxidative stress [172]. A- and β-Pinene are the main constituents of gum essential oil of *P. atlantica*. Administration of the essential oil to diabetic rats caused a significant decrease in MDA and increase in glutathione, glutathione peroxidase, superoxide dismutase, and catalase [173]. In a recent study, in vitro α-amylase enzymatic assay has shown that both α-pinene (IC_50_ 1.05 ± 0.0252 mg mL^−1^) and β-pinene (IC_50_ 1.17 ± 0.0233 mg mL^−1^) resulted in a 32% and 29% drop in enzymatic activity respectively [145].

#### 4.3.2. Thujone

Thujone [(1S,4R,5R)-4-methyl-1-propan-2-yl)bicyclo[3.1.0]hexan-3-one] occurs mainly as a mixture of α and β diastereoisomers in many plants including *Salvia officinalis* L. (sage), *Artemisia absinthium* L., and *Thuja occidantalis* L. Traditionally, it was used by native Americans as a remedy for several ailments such as headache, constipation, wounds, and birthmarks. This monoterpene is commonly used as a flavoring substance in food and beverages [174]. Interestingly, sage tea is known for its metformin-like effect, in particular for the essential oil fraction which contains thujone. Therefore, thujone could possibly exhibit some sort of an antidiabetic effect [175]. Nevertheless, animal studies that have pointed to the potential antidiabetic activity of thujone are limited. For example, in soleus muscles, palmitate-induced insulin resistance was assessed in the presence of thujone (0.01 mg/mL). Initially, insulin resistance was induced with high concentrations of palmitate [176]. Subsequently, the ability of thujone to restore sensitivity to insulin while preserving high palmitate concentrations was tested. The findings of this study indicated that thujone can ameliorate palmitate oxidation and prevent palmitate-induced insulin resistance via AMP-activated protein kinase (AMPK)-dependent pathway that involves partial restoration of insulin-stimulated translocation of GLUT4 [177]. Al-Haj Baddar, et al., 2011 demonstrated that oral administration of 5 mg/kg body weight of thujone in diabetic rats over 28 days can restore the normal levels of cholesterol and triglycerides [175]. While this finding is promising, the adverse effects of thujone necessitates careful analysis of the results. The narrow therapeutic window of thujone is evident in 2-year studies in rats and mice due to the dose-dependent incidence of seizures [178].

#### 4.3.3. Myrtenal

Myrtenal [6,6-dimethylbicyclo[3.1.1]hept-2-ene-2-carbaldehyde] is a natural monoterpene present in plants such as pepper, mint, cumin, and eucalyptus and used as a food additive. It has various biological effects and acts as an antioxidant, anticancer agent, cyclooxygenase-inhibitor, and immunostimulant [179,180]. Recently, it was found that myrtenal exhibits antihyperglycemic, antihyperlipidemic, hepatoprotective, and β-cell protective effects [181,182].

Oral treatment with myrtenal (20, 40, and 80 mg/kg body weight) resulted in a significant depletion in plasma glucose and HbA1c in diabetic rats treated with streptozotocin. Additionally, there was a rise in insulin, hemoglobin (Hb), and glycogen levels in the liver and muscles. An enhancement of the main enzymes involved in carbohydrate metabolism (hexokinase, glucose-6-phosphatase, fructose-1,6-bisphosphatase, and glucose-6-phosphate dehydrogenase) was observed. Furthermore, myrtenal enhanced hepatic enzyme function and restored islet cells and liver histology [182].

In parallel to the above findings, another study has shown that myrtenal-treated diabetic rats displayed a reduction in plasma glucose and a simultaneous rise in plasma insulin. Additionally, myrtenal caused an upregulation in the expression of proteins involved in insulin signaling such as IRS2 (insulin receptor substrate 2), Akt, and GLUT2 in hepatocytes as well as IRS2, Akt, and GLUT4 in skeletal muscle [183].

Recently, the influence of myrtenal on oxidative stress, inflammation, and lipid peroxidation was tested on diabetic rats treated with streptozotocin. Oral administration of 80 mg/kg body weight of myrtenal for four weeks significantly decreased the diabetes-associated alterations in hepatic and pancreatic cells. This includes antioxidant levels, lipid peroxidation, and proinflammatory cytokines such as TNF-α, IL-6, and the p65 subunit of nuclear factor-kappa B (NF-kB p65). The findings of this work indicated that myrtenal can potentially act as an antioxidant and anti-inflammatory compound against oxidative stress and inflammation associated with diabetes [184].

#### 4.3.4. Genipin and Geniposide

The iridoids genipin [methyl-1-hydroxy-7-(hydrozymethyl)-1,4a,5,7 tetrahydrocyclopenta[c]pyran-4-carboxylate] and geniposide [methyl (1S,4aS,7aS)-7-(hydroxymethyl)-1-[(2S,3R,4S,5S,6R)-3,4,5-trihydroxy-6-(hydroxymethyl)oxan-2-yl]oxy-1,4a,5,7a tetrahydrocyclopenta[c]pyran-4-carboxylate] exist in many plants as secondary metabolites. The basic structural skeleton of iridoids is a cyclopentane-[C]-pyran ring fused with a six-membered heterocycle oxygenate [185]. At C1 position of the pyran ring, the hydroxyl group can be replaced with a sugar moiety to form the genipin glycoside, geniposide. Genipin is found in unripe *Genipa americana* L. (genipa) fruits, while geniposide is found in the fruits of *Gardenia jasminoides* J. (gardenia, Rubiaceae family) that has been used in traditional Chinese medicine for its choleretic and hepatoprotective activity. Earlier studies have shown that geniposide is converted to genipin by the intestinal microflora enzymes, which indicates that genipin is the main form of geniposide in circulating blood [186].

Genipin was shown to have anticancer, anti-inflammatory, hepatoprotective as well as antioxidative activity [187]. Geniposide exhibits many biological effects including antioxidative stress [188], anti-inflammatory [189] and antiapoptosis [190]. In addition, studies have shown that it exerts a promising anti-diabetic activity. For example, in C(2)C(12) myotubes, genipin (10 μM) stimulated glucose uptake in a time- and concentration-dependent manner. It also enhanced GLUT4 translocation to the cell surface and increased the phosphorylation of IRS-1, AKT, and GSK3β. Genipin also caused a rise in ATP levels, which inhibited ATP-dependent K^+^ channels and resulted in elevated cytoplasmic Ca^2+^ content [191]. 

Administration of 25 mg/kg of genipin per day for 12 days to aged rats ameliorated systemic as well as hepatic insulin resistance. It also alleviated hyperinsulinemia, hyperglyceridemia, and hepatic steatosis. Furthermore, genepin reduced hepatic oxidative stress as well as mitochondrial dysfunction. It also improved insulin sensitivity, suppressed cellular ROS overproduction, and alleviated the reduction in mitochondrial membrane potential (MMP) and ATP levels [192]. Guan et al., 2018 studied the effect of genipin on obesity and lipid metabolism in diet-induced obese rats. The findings of this study demonstrated that genipin caused an overall drop in body weight and total fat. Additionally, it reversed insulin and glucose intolerance, dyslipidemia, adipocyte hypertrophy, and hepatic steatosis. It also caused a reduction in serum TNF-α levels [193]. Similar results were reported by Zhong et al., 2018, where genipin alleviated hyperlipidemia and hepatic steatosis in high-fat diet fed mice [194].

Earlier study has shown that geniposide exhibits anti-obesity, anti-oxidant, and insulin resistance-alleviating effects. Additionally, it was shown to adjust abnormal lipid metabolism. In spontaneously obese T2DM TSOD mice, geniposide caused a reduction in visceral fat and body weight and improved lipid metabolism. Furthermore, geniposide had a positive therapeutic impact on glucose tolerance and hyperinsulinemia. Interestingly, geniposide had a direct effect on the liver. In mice treated with free fatty acids, genipin not only inhibited lipid accumulation hepatocytes, but also improved the expression of PPARα [195]. 

Emerging body of evidence revealed that lipotoxicity may be a leading cause of pancreatic β-cell apoptosis and oxidative stress in diabetes. Increased levels of plasma-free fatty acids not only induce cytotoxicity in pancreatic β-cells leading to apoptosis, but also promote mitochondrial perturbation, resulting in oxidative stress. In pancreatic INS-1 cells, application of geniposide (1 or 10 μM) for 7 h alleviated β-cell apoptosis induced by palmitate and activated caspase-3 expression. Furthermore, geniposide improved glucose-induced insulin secretion via the activation of GLP-1 receptor [196]. Another study has demonstrated that when INS-1 cells are chronically exposed to elevated glucose concentrations, insulin secretion was impaired and cell apoptosis was observed. This change was reversed by the application of geniposide [197]. However, the effects of geniposide on insulin secretion after acute exposure to glucose was dependent on glucose concentration. When INS-1 cells were acutely stimulated with high glucose concentrations, the protective effect of geniposide was diminished. This could be attributed to the capability of geniposide to protect the cells from damage resulting from prolonged release of insulin and glucotoxicity under high glucose load [198].

An earlier study has assessed the direct effect of geniposide on β-cell function using both rat pancreatic islets and dispersed single islet cells [199]. Geniposide was found to mediate insulin release via the activation of GLP-1R and adenylyl cyclase (AC)/cAMP signaling pathway. In general, the effect of GLP-1R agonists is linked to cAMP signaling [200]. In this study, PKA suppression inhibited geniposide-mediated secretion of insulin, implying that geniposide exhibited its actions mainly via the activation of cAMP-dependent PKA [199]. It is well known that activation of pancreatic voltage-gated K^+^ channels repolarizes cells and suppresses insulin release. Therefore, inhibition of these channels could prolong the duration of the action potential and promote glucose-dependent insulin secretion [201]. Interestingly, Zhang et al., 2016 stated that geniposide can inhibit voltage-gated K^+^ channels in a concentration-dependent manner. This was diminished upon treating β-cells with GLP-1R and PKA inhibitors. Collectively, the findings of this study suggest that inhibition of voltage-gated K^+^ channels is coupled to geniposide-induced insulin release by activating the downstream of GLP-1/cAMP/PKA signaling pathway [199]. 

#### 4.3.5. Catalpol

Catalpol[(2S,3R,4S,5S,6R)-2-[[(1S,2S,4S,5S,6R,10S)-5-hydroxy-2-2(hydroxymethyl)-3,9-dioxatricyclo[4.4.0.0^2,4^]dec-7-en-10-yl]oxy]-6-(hydroxymethyl) oxane-3,4,5-triol, is an iridoid glucoside isolated from the root of *Rehmannia glutinosa*, which has previously been used in traditional Chinese medicine to manage hyperglycemia for decades. Earlier studies have reported that catalpol exhibits an antidiabetic potential, which is attributed to its antioxidant property. In animal models, the oral dose of catalpol that caused a significant antidiabetic effect ranged from 2.5 to 200 mg/kg and 10 to 200 mg/kg in rats and in mice, respectively [202].

Catalpol acts through several mechanisms that affect insulin-sensitive organs like the liver, skeletal muscle, adipose tissue, and pancreas. Furthermore, catalpol adjusts several genes and proteins in the pancreas, skeletal muscle, and adipose tissue that have a crucial role in the management of diabetes [202].

In high-fat and streptozotocin-treated diabetic C57BL/6J mice, administration of 100 and 200 mg/kg catalpol over four weeks decreased the p (Ser 307)-IRS-1 and increased the p (Ser 347)-AKT and p (Ser 9)-GSK3 β. Such effect adjusted the impaired insulin pathway in the liver through PI3K/AKT pathway. Furthermore, catalpol prevented gluconeogenesis by enhancing the activity of AMPK and inhibiting PEPCK and G6Pase protein expression [203]. In spontaneous diabetic db/db mice treated with 80 or 160 mg/kg catalpol for four weeks, p-AMPK and GLUT expression were significantly enhanced in liver, skeletal muscle, as well as adipose tissue, which promoted the uptake of glucose into the cells [204]. 

In spontaneous diabetic db/db mice, the lowered expression of IRS-1 resulted in negative regulation of insulin signaling cascades, as IRS-1 is an important ligand in activating the PI3K/AKT pathway. Furthermore, decreased activity of isocitrate dehydrogenase 2 (IDH2), an enzyme that catalyzes the citrate cycle, attenuates glucose metabolism and ATP production. It is well-known that glucose-6-phosphate 1-dehydrogenase (G6PD2) catalyzes the pentose phosphate pathway that utilizes glucose to produce NADPH and ribose-5-phosphate. The downregulation of G6PD2 enzyme decreases the glucose metabolism. On the other hand, upregulation of suppressor of cytokine signaling 3 (SOCS3) enzyme can inhibit the tyrosine phosphorylation of the insulin receptor, leading to the suppression of insulin signaling pathway [205,206,207]. Liu et al., 2018 reported that oral treatment with catalpol (25, 50, 100, and 200 mg/kg) upregulated IRS-1, IDH2, and G6PD2 expression, and downregulated SOCS3. Collectively, the findings indicate that catalpol can increase glucose metabolism through accelerating the citrate cycle and pentose phosphate pathway and promoting insulin signaling pathway [204]. 

**Figure 4:**
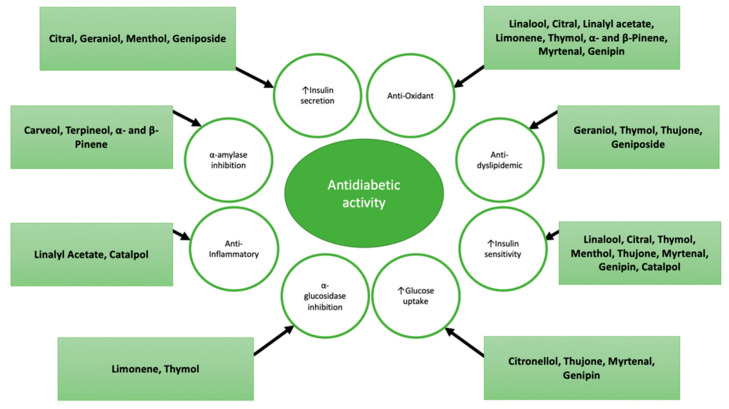
Mechanisms of action of different monoterpenes.

The antidiabetic effects of bicyclic monoterpenes are summarized in Table 3. The mechanisms of action of the above-mentioned monoterpenes are summarized in Figure 4.

## 5. Structure–Activity Relationship

Although monoterpenes possess multiple pharmacological and molecular mechanisms of action, their structure–activity relationship has not been fully elucidated yet. In vitro and in vivo data summarized in this review demonstrate that there is a wide range of mechanisms of action by which monoterpenes exhibit their antidiabetic effects. These include (1) inhibition of α-amylase and α-glucosidase, (2) stimulation of insulin release, (3) stimulation of glucose uptake, (4) increase in insulin sensitivity, (5) inhibition of gluconeogenesis, (6) reduction in cellular oxidative stress, (7) reversal of dyslipidemia, (8) increase in anti-inflammatory activity, and (9) inhibition of pancreatic β-cell apoptosis. The current review discusses the antidiabetic effect of different monoterpenes using in vitro, as well as in vivo models, in which oxidative metabolism is an essential factor to consider. For example, *p*-cymene could be hydroxylated as a result of oxidative metabolism at a position comparable to the hydroxyl group position in α-terpineol. Hydroxylation of *p*-cymene also leads to the biosynthesis of an entirely different monoterpene, namely thymol, in which the antioxidant and antidiabetic properties are attributed to the pharmacophore of the phenolic hydroxyl group in its chemical structure. Therefore, it could be highly anticipated that structural modification of the parent molecule (*p*-cymene), such as the introduction of hydroxyl group, enhances its antioxidant activity. This is also applicable to other compounds, such as citral, which contains an aldehyde group. It is well-known that aldehydes are highly resistant to oxidative deterioration [208]. Citral has a high tendency to be oxidized and therefore, the aldehyde group could be easily converted to a carboxylic acid group. Such potential metabolism of the aldehyde group is also applicable to the compound myrtenal. Moreover, limonene is a precursor for carveol. Considering the carbon numbering relative to limonene, the presence of an oxygenated group at carbon-6 conjugated to a double bond at carbon-1 and an isopropenyl group at carbon-4 were found to be the major chemical features relevant for activity and potency of carveol. For example, compared to limonene and other limonene derivatives, carveol significantly decreased lipopolysacharide (LPS)-induced nitric oxide (NO) production in murine macrophages. This anti-inflammatory activity was credited to the chemical features that are absent in other compounds [209]. Earlier studies have attributed the effect of monoterpenes to their volatility [11], hydrophobicity [210], and non-specific [211] and non-competitive [212] mechanisms of action. The lipophilic characteristic of the monoterpene skeleton combined with the nature of the functional group is essential for its activity. It has been proposed that the rank of activity is the greatest for aldehydes (e.g., citral), followed by alcohols (e.g., linalool and geraniol), followed by hydrocarbons (e.g., p-cymene and limonene). It should also be noted that some monoterpenes (e.g., catalpol) that exist in glycosylated form are very polar, which also affects their biological activity [213]. Compounds that contain phenolic groups are known to confer protection against the deleterious effects of free radicals both by absorbing or neutralizing free radicals and by augmenting endogenous antioxidants [214]. Additionally, studies have shown that the presence of a phenolic structural moiety displays potent antioxidant effects and/or direct radical scavenging that can account for the antidiabetic activity of monoterpenes. Thymol and 4-terpineol are typical examples that have been reported for their antihyperglycemic effects [145,154]. Supporting these findings, Zunino and Zygadlo (2004) concluded that most potent monoterpenes are those that are alcohols and phenols [215]. A study conducted by Javan and Javan (2014) evaluated the structure-radical scavenging activity of thymol derivatives. It was concluded that the presence of an unsaturated double bond is the main factor that determines the antioxidant and radical scavenging activity of the monoterpene derivatives [216]. Interestingly, it was shown that the incorporation of monoterpenes into other groups such as flavonoids augments their antioxidant effect [217]. Whether a monoterpene is a simple hydrocarbon (e.g., *p*-cymene and limonene), hydroxy derivative, or phenolic, a potential antidiabetic effect has been reported at low doses. However, due to the wide range of variations in experimental settings (e.g., range of concentrations tested, modes of drug administration, cell type, and animal models used), in addition to controversial in vitro and in vivo findings and their species dependency, direct comparison of in vitro and in vivo potency between the various subtypes of monoterpenes is difficult. In fact, more in vivo studies should be undertaken to confirm in vitro findings. Furthermore, a full-scale pharmacokinetic profiling is needed to interpret the inconsistency between results observed in in vitro and in vivo preclinical studies. 

Based on the above, structure–activity relationship among monoterpenes can be made only when the effect of each compound (acyclic, monocyclic, and bicyclic) is investigated using a single target in vitro, in which pharmacokinetic profile (absorption, distribution, metabolism, and elimination) is excluded. In addition, an in silico molecular docking approach must be used to predict the molecular mechanism of action of each monoterpene on its potential target related to diabetes. Determination of the order of potency of the monoterpenes under standardized conditions, will help in correlating the activity with structural features to identify the relevant structural determinants of antidiabetic activity.

## 6. Summary and Conclusions

DM is a disease associated with high rates of morbidity and mortality and one of the leading causes of death in the world. The major complications associated with diabetes mellitus are classified as microvascular (including retinopathy, neuropathy, and nephropathy) and macrovascular (including cardiovascular myopathy and cerebrovascular diseases) [218,219]. Hyperglycemia plays an important role in the onset and development of these complications, mainly by generating reactive oxygen species (ROS) which causes lipid peroxidation and membrane damage. Cardiovascular (CV) risk factors such as obesity, hypertension, and dyslipidemia are common in patients with DM, placing them at increased risk for cardiac events. DM can be controlled by targeting multiple components like glucose transport, insulin signaling, insulin secretion, lipid regulation, inflammation, and oxidation. Despite the availability of different classes of antidiabetic agents, side effects like weight gain and hypoglycemia affect patients’ adherence to therapy. Novel medicinal compounds can be synthesized and designed for the treatment of several diseases based on the chemical structure of these molecules. Monoterpenes are the main components of essential oils and have been recognized for their wide range of cellular and molecular activities that could potentially underlie their positive therapeutic index. Due to their abundance in occurrence, various biological activities, and high safety profile, monoterpenes became central for research and development around the globe. In this article, the pathogenesis of DM and the classes of antidiabetic agents used for the management of the disease were discussed. Moreover, we summarized the effects of selected acyclic, monocyclic, and bicyclic monoterpenes that are supposed to possess a potential role in the management of DM. Based on the fact that monoterpenes show structural complexity and diversity, comparison of the net antidiabetic effect between the three subcategories of monoterpenes cannot be made due to inconsistency in dose, duration, mode of drug administration, target tissue, and animal model used. To accurately determine which category of monoterpenes (acyclic, monocyclic, bicyclic) can exhibit the greatest antidiabetic effect, a comparison must be made using the exact same experimental conditions (concentration used, cell and tissue type targeted, etc.). However, based on extensive review of experimental studies, it has been proposed that the rank of activity is the greatest for aldehydes (e.g., the acyclic monoterpene citral), followed by alcohols (e.g., the acyclic monoterpenes linalool and geraniol), followed by hydrocarbons (e.g., the monocyclic monoterpenes p-cymene and limonene) [213]. Due to the fact that monoterpenes provide a promising area of research, further studies with regards to their structure-activity relationship as well as structural modification are crucial to maximize their therapeutic effects. Their use in combination with other monoterpenes or natural compounds should be carried out in the future to fill in the gaps. Additionally, more research is still needed to investigate the actions of these molecules on diabetic patients in order to confirm their therapeutic ability in controlling hyperglycemia and dyslipidemia caused by the disease.

## Figures and Tables

**Table 1:** Antidiabetic effects of acyclic monoterpenes.

Compound	Model	Concentration	Antidiabetic Activities	References
Linalool	T2DM rat model	Tea preparation (0.25 g/100 mL and 0.5 g/100 mL for 4 weeks)	Lowered serum glucose and lipids; increased insulin sensitivity and levels of serum insulin; improved β-cell function, increased liver glycogen	[106]
	Diaphragm of streptozotocin-induced diabetic rat	3 mM	Decreased oxidative stress, increased the activity of the antioxidant enzymes catalase and superoxide dismutase.	[105]
Citral	Hemi diaphragm of Albino rat	3 mM	Increased glucose uptake	[105]
	Streptozotocin-induced diabetic rats	2, 8, 16 or 32 mg/kg body weight	Inhibited adipogenesis; increased metabolic rate, reduced weight gain; enhanced glucose tolerance.	[112]
	Streptozotocin-induced diabetic rats	2, 8, 16 or 32 mg/kg body weight	Inhibition of α-amylase.	[112]
	3T3-L1 adipocytes	1 μM	Suppression of adipocyte proliferation of by 29.2%.	[98]
	6-week-old male Sprague–Dawley rats	10, 15, and 20 mg/kg body weight for 28 days	Increased energy dissipation; reduced lipid accumulation; prevention of diet-induced obesity; improved insulin sensitivity and glucose tolerance.	[119]
	Streptozotocin-induced diabetic rats fed with high-fat diet	45 mg/kg/body weight for 28 days	Decreased blood glucose and increased plasma insulin; increased anti-oxidative enzymes of the liver, adipose tissue, and pancreas; regulated enzyme activity of glycolysis and gluconeogenesis in the liver.	[120]
Geraniol	Streptozotocin-induced diabetic rats	100, 200, 400 mg/kg body weight for 45 days	Increased the levels of insulin and hemoglobin; decreased plasma glucose HbA1c; ameliorated carbohydrate metabolism; preserved normal histological appearance of hepatic and pancreatic β-cells.	[122]
		648.34 μM	Inhibited GLUT2 transporter.	[123]
		60 days with 29.37 mm/kg B.W. twice a day	Improved lipid profile, HbA1c levels and renal parameters.	[123]
Citronellol	Streptozotocin-induced diabetic rats	Oral administration of 25, 50, and 100 mg/kg body weight for 30 days	Improved levels of insulin, hemoglobin, and hepatic glycogen; decreased levels of HbA1c_;_ restored altered activities of carbohydrate metabolic enzymes, hepatic and kidney markers; preserved normal histological appearance of hepatic cells and insulin-positive β-cells	[124]
	3T3-L1 adipocytes	1 μM	Enhanced glucose uptake	[98]
Linalyl acetate	Streptozotocin-induced diabetic rats	100 mg/kg	Decreased serum glucose; reduced oxidative stress and inflammation	[132]

**Table 2:** Antidiabetic effects of monocyclic monoterpenes.

Compound	Model	Concentration	Antidiabetic Activities	References
Limonene	Streptozotocin-induced diabetic rats	50 µM and 100 µM	Inhibited protein glycation.	[105,135]
	Streptozotocin-induced diabetic rat	100 µM	Increased activity of catalase and superoxide dismutase.	[105]
	3T3-L1 adipocytes	1 µM	Increased glucose uptake and lipolysis; upregulated mRNA expression GLUT1 and suppressed ATGL.	[98]
		mM range	Inhibited α-amylase and α-glucosidase	[98]
		50 mg/kg body weight	Decreased DNA damage, decreased glutathione reductase enzyme activity, decreased the levels of MDA in the plasma; increased total glutathione levels, catalase, superoxide dismutase and glutathione peroxidase activities	[134]
		50, 100 and 200 mg/kg body weight and for 45 days	Increased plasma glucose, HbA1c levels, and activities of gluconeogenic enzymes; decreased the activity of glucokinase.	[136]
Carveol	Alloxan-induced diabetic rat	394.1 µM/kg	Improved oral glucose tolerance overload in; decreased the level of HbA1c; inhibited α-amylase activity.	[8]
Terpineol	α-amylase enzymatic assay	α-terpineol 0.670 mg/mL 4-terpineol 0.670 mg/mL	Inhibited α-amylase activity Inhibited α-amylase activity	[145]
Thymol	High-fat diet induced T2DM in C57BL/6J mice	Intragastric administration of 40 mg/kg body weight daily for 5 weeks.	Decreased plasma glucose, insulin resistance, HbA1c, leptin and adiponectin; lowered the levels of plasma triglyceride, total cholesterol, free fatty acids, low density lipoprotein; increased high density lipoprotein cholesterol; decreased in hepatic lipid content.	[154]
	C57BL/6J mice	40 mg/kg body weight daily for 5 weeks	Protected against diabetic nephropathy; inhibited the activation of transforming growth factor-β1 (TGF-β1) and vascular endothelial growth factor (VEGF), elevated antioxidants, inhibited lipid peroxidation markers in erythrocytes and kidney tissue, reduced the lipid accumulation in kidney	[156]
	High-fat diet-induced obesity in murine model	14 mg/kg orally twice a day to 4 weeks	Decreased body weight gain, visceral fat-pad weights, lipids, alanine aminotransferase, aspartate aminotransaminase, lactate dehydrogenase, glucose, insulin, and leptin levels	[152]
	Streptozotocin-induced diabetic rats	20 and 40 mg/kg thymol	Reduced creatinine, low-density lipoprotein cholesterol, and liver function-related enzymes, aspartate aminotransferase and alanine aminotransferase	[157]
	1,1-dephenyl-2-picryl-hydrazyl free radical scavenging and a reducing power assay		Increased radical scavenging activity	[158]
	In vitro α-glucosidase assay		Decreased α-glucosidase activity	[158]
*p*-Cymene	High-fat diet fed adult NMRI mice	20 mg/kg body weight for 6 weeks	Decreased levels of blood glucose, alanine aminotransferase and alkaline phosphatase; altered lipid profile.	[163]
	Streptozotocin-induced diabetic rat	20 mg/kg body weight for 60 days	Lowered HbA1c, prevented glycation-mediated transition of α-helix to β-pleated sheet structure of bovine serum albumin.	[164]
Menthol	High-fat diet fed mice	Acute oral (200 mg/kg) and topical administration (10% *w*/*v*)	Increased serum glucagon concentration;	[168]
		Chronic oral administration (50 and 100 mg/kg/day for 12 weeks) and topical Application (10% *w*/*v*)	Prevented high fat diet-induced weight gain, insulin resistance, adipose tissue hypertrophy and triacylglycerol deposition in liver.	[168]
	Mature 3T3L1 adipocytes treated with serum of menthol-treated mice in	0.3 μM	Improved energy expenditure markers, which was blocked in the presence of non-competitive glucagon receptor antagonist, L-168,049.	[168]
	Streptozotocin-nicotinamide -induced diabetic rats	25, 50, and 100 mg/kg/body weight for 45 days	Reduced the level of blood glucose and HbA1c; increased the level of total hemoglobin, plasma insulin, and liver glycogen.	[167]

**Table 3:** Antidiabetic effects of bicyclic monoterpenes.

Compound	Model	Concentration	Antidiabetic Activities	References
α-Pinene	Alloxan-induced diabetic mice	i.p. injection of 0.25 mL/kg α-pinene	Evoked hypoglycemia activity at the 2nd and 24th hours.	[10]
	α-amylase enzymatic assay	0.670 mg/mL	Inhibited α-amylase activity.	[145]
β-Pinene	Streptozotocin-induced diabetic rat	Oral administration of 30 mg/kg of hexanic extract (17.53% β-pinene) daily for 7 weeks	Ameliorated hyperglycemia and oxidative damage.	[170]
	α-amylase enzymatic assay	0.670 mg/mL	Inhibited α-amylase activity.	[145]
Thujone	Palmitate-induced insulin resistance in soleus muscles of male Sprague-Dawley rats	0.01 mg/mL (incubation for 6 h in presence of palmitate)	Restored insulin sensitivity; ameliorated palmitate oxidation and rescued palmitate-induced insulin resistance via AMPK-dependent mechanism involving partial restoration of insulin-stimulated GLUT4 translocation.	[177]
	Alloxan monohydrate-induced diabetic rats	5 mg/kg thujone for 28 days	Adjusted cholesterol and triglyceride levels to normal levels.	[175]
Myrtenal	Streptozotocin-induced diabetic rat	80 mg/kg body weight (orally)	Adjusted antioxidant levels, lipid peroxidation, and proinflammatory cytokines (TNF-α, IL-6, NF-kB p65).	[184]
	Streptozotocin-induced diabetic rat	80 mg/kg body weight (orally)	Reduced plasma glucose; increased plasma insulin; upregulated IRS2, Akt, and GLUT2 in hepatocytes; upregulated IRS2, Akt, and GLUT4 in skeletal muscle.	[183]
	Streptozotocin-induced diabetic rat	20, 40, and 80 mg/kg body weight (orally)	Depleted plasma glucose and HbA1c; increased insulin, Hb, and hepatic and muscle glycogen; enhanced carbohydrate metabolic enzymes and hepatic enzyme function; restored islet cells and liver histology.	[182]
Genipin	C2C12 myotubes	10 μM	Promoted GLUT4 translocation to the cell surface; increased the phosphorylation of IRS-1, AKT, and GSK3β; increased ATP levels which inhibited ATP-dependent potassium channels; increased cytoplasmic calcium.	[191]
	Aging rats	25 mg/kg genipin or vehicle once daily for 12 days	Adjusted insulin resistance; ameliorated systemic and hepatic insulin resistance; alleviated hyperinsulinemia, hyperglyceridemia, and hepatic steatosis; reduced hepatic oxidative stress and mitochondrial dysfunction; improved insulin sensitivity; inhibited cellular ROS overproduction; alleviated the reduction of levels of MMP and ATP.	[192]
	Diet-induced obese rats		Reduced body fat; Reversed dyslipidemia, glucose and insulin intolerance, adipocyte hypertrophy, and hepatic steatosis. Reduced serum tumor necrosis factor-α levels.	[193]
	Diet-induced obese mice	5 or 20 mg/kg/day	Alleviated high-fat diet induced hyperlipidemia and hepatic steatosis.	[194]
Geniposide	Spontaneously obese T2DM TSOD mice		Caused a reduction in body weight and visceral fat accumulation, improved lipid metabolism and intrahepatic lipid accumulation, adjusted hyperinsulinemia glucose tolerance, inhibited the accumulation of lipid in hepatocytes of free fatty acid treated rats, improved the expression of PPAR*α*.	[195]
	Pancreatic INS-1 cells	1 or 10 μM for 7 h	Alleviated β-cell apoptosis induced by palmitate, activated caspase-3 expression, improved glucose stimulated insulin secretion by activating GLP-1R	[198]
	Pancreatic INS-1 cells	1 or 10 μM for 5 days	Increased insulin secretion in β-cells and decreased apoptosis	[197]
	Pancreatic islets and dispersed single islet cells from Male Sprague- Dawley (SD) rat	1 and 10 μM	Inhibition of voltage-dependent potassium, activated GLP-1/cAMP/PKA signaling pathway and insulin secretion.	[199]
Catalpol	High-fat diet and streptozotocin-induced diabetic C57BL/6J mice	100 or 200 mg/kg, p.o., four weeks	Adjusted the impaired insulin pathway in the liver through PI3K/AKT pathway (decreased p (Ser 307)-IRS-1 and increased the p (Ser 347)-AKT and p (Ser 9)-GSK3 β), prevented gluconeogenesis by enhancing the activity of AMPK and inhibiting PEPCK and glucose G6Pase protein expression.	[203]
	db/db mice	25, 50, 100, and 200 mg/kg (orally)	Upregulated the expression of IRS-1, IDH2, and G6PD2, and downregulated the expression of the SOCS3.	[205]
	High fat diet and streptozotocin-induced diabetic mice	100 or 200 mg/kg for four weeks (orally)	Upregulated SOD2 and GSH-Px, suppressed the serum level of MDA and NOX4.	[203]
	Glucosamine-treated HepG2 cells	20–80 µM	Increased the levels of SOD and GSH-Px, decreased the MDA level and NOX4 protein expression.	[203]
	C57BL6/J mice fed with high fat diet	200 mg/kg for 4–8 weeks	Increased skeletal muscle insulin sensitivity by activating IRS-1/AKT/GLUT4.	[203]
	db/db mice	200 mg/kg for 8 weeks	Augmented myogenesis by increasing expression of MyoD, MyoG and MHC expressions	[204]
	High glucose treated C2C12 cells	10, 30, 100 µM for 24 h	Increased MyoD and MyoG mRNA/protein levels.	[203]
	Skeletal muscle of db/db mice	200 mg/kg/day for 8 weeks (orally)	Increased number of mitochondria, mitochondrial DNA levels, and expression of genes involved in mitochondrial biogenesis.	[205]
